# Risk factors for delayed neurocognitive recovery in elderly patients undergoing thoracic surgery

**DOI:** 10.1186/s12871-023-02056-6

**Published:** 2023-03-31

**Authors:** Lu Wang, Bin Chen, Tao Liu, Taijun Luo, Wanli Kang, Wei Liu

**Affiliations:** 1 Beijing Tuberculosis and Thoracic Tumor Research Institute, Beijing, China; 2 Department of Anesthesiology, Beijing Chest Hospital, Capital Medical University, Beijing, China; 3 Department of Anesthesiology, Beijing Jishuitan Hospital, Beijing, China; 4 Department for disease prevention and control, Beijing Chest Hospital, Capital Medical University, Beijing, China

**Keywords:** *Perioperative neurocognitive disorders*, *Delayed neurocognitive recovery*, *Thoracic surgery*, *Elderly patients*

## Abstract

**Background:**

To investigate the risk factors for delayed neurocognitive recovery in elderly patients undergoing thoracic surgery.

**Methods:**

A total of 215 elderly patients who underwent thoracic surgery between May 2022 and October 2022 were recruited in this prospective observational study. Cognitive function was tested by MoCA tests that were performed by the same trained physician before surgery, on postoperative day 4 (POD4), and on postoperative day 30 (POD30). Univariate and multivariate logistic regression models were used to analyze the risk factors for DNR.

**Results:**

A total of 154 patients (55.8% men) with an average age of 67.99 ± 3.88 years were finally included. Patients had an average preoperative MoCA score of 24.68 ± 2.75. On the 30th day after surgery, 26 (16.88%) patients had delayed postoperative cognitive recovery, and 128 (83.12%) had postoperative cognitive function recovery. Diabetes mellitus (OR = 6.508 [2.049–20.664], P = 0.001), perioperative inadvertent hypothermia (< 35℃) (OR = 5.688 [1.693–19.109], P = 0.005), history of cerebrovascular events (OR = 10.211 [2.842–36.688], P < 0.001), and VICA (sevoflurane combined with propofol anesthesia) (OR = 5.306 [1.272–22.138], P = 0.022) resulted as independent risk factors of delayed neurocognitive recovery. On the POD4, DNR was found in 61 cases (39.6%), and age ≥ 70 years (OR = 2.311 [1.096–4.876], P = 0.028) and preoperative NLR ≥ 2.5 (OR = 0.428 [0.188–0.975], P = 0.043) were identified as independent risk factors.

**Conclusions:**

The risk factors for delayed neurocognitive recovery in elderly patients undergoing thoracic surgery include diabetes, perioperative inadvertent hypothermia (< 35℃), VICA (sevoflurane combined with propofol anesthesia), and history of cerebrovascular events.

## Background

Delayed neurocognitive recovery (DNR) is defined as a cognitive decline that occurs within 30 days of surgery. It is a common postoperative complication in elderly patients characterized by impairments in memory and attention, usually detected by neuropsychological tests [1]. Delayed postoperative neurocognitive recovery (DNR) occurs in 15–50% of elderly surgical patients on the day of hospital discharge [2–4]. Previous studies have shown that, in elderly patients undergoing thoracic surgery, the occurrence of DNR within 1 week after surgery is 20–60% [3, 5]; however, there are very few studies on the incidence of cognitive recovery delay in elderly patients who underwent thoracic surgery at 30 days after surgery.

Thus far, DNR has been associated with several perioperative complications, resulting in a prolonged hospital stay. As such, it constitutes a major public health concern considering that patients with DNR at discharge were found to have increased mortality at 3 months or 1 year after surgery. It also poses a great economic burden to society [6]. Identifying potential DNR risk factors is of utmost importance to facilitate risk stratification and preventive efforts. The clinical relevance of delayed neurocognitive recovery after thoracic surgery requires more attention.

Although several studies have reported various risk factors for DNR after non-cardiac major surgery, the risk factors for DNR in elderly patients undergoing thoracic surgery are still poorly understood [2, 7]. Thoracic surgery often requires one-lung ventilation, which is accompanied with important physiological disturbances, and leads to a pulmonary arteriovenous shunt with the decrease of arterial oxygen content and an exaggerated activation of inflammatory processes [8]. Furthermore, postoperative pain resulting from thoracic surgery is usually more severe than the pain caused by other major surgery and is influenced by neuropathic pain caused by thoracotomy [9]. Systemic inflammatory response and severe persistent pain after surgery causes cognitive negative events [10]. Besides, most previous studies evaluated the cognitive function within 1 week after surgery; patients were only followed up until discharge, and there was a lack of long-term follow-up. In the present study, we planned to follow up with participants at POD30. The risk factors for DNR could vary for patients undergoing different types of major surgery. The purpose of this study was to investigate risk factors for DNR in elderly patients after thoracic surgery.

## Methods

### Study design and population

Elderly patients who underwent thoracic surgery with general anesthesia in Beijing Chest Hospital between May and October, 2022 were prospectively enrolled in this study. All patients signed informed consent prior to surgery. Our institutional follow-up protocol included postoperative cognitive assessment at POD4 and POD30, and our primary endpoint is the incidence rate of DNR at POD30. The study was approved by the Ethics Committee of Beijing Chest Hospital (ID: YJS-2022-02) and registered in the China Clinical Trial Center (ChiCTR2200061670).

Inclusion criteria were the following: age ≥ 60 years; preoperative Montreal Cognitive Assessment (MoCA) test score ≥ 18; plan to have elective thoracic surgery. Exclusion criteria were: significant sequelae of cerebral infarction; severe central nervous system disease or mental illness; history of drug or alcohol abuse; severe visual or hearing impairment; illiteracy; thoracotomy with pneumonectomy or median sternum split; postoperative respiratory failure (postoperative mechanical ventilation for more than 48 h or unplanned reintubation); reoperation within 30 days.

### Cognitive function measurement

A trained investigator tested patients in a quiet room. The interviewer underwent training on how to conduct brief structured cognitive assessments of attention, orientation, and memory. The baseline test included the MoCA test Beijing version (from http://www.mocatest.org) for assessment of the cognitive level of the patients the day before surgery. The first postoperative cognitive test was performed on day 4 after surgery, and the MoCA test Mandarin version 7.2 (the translated version of MoCA-8.2-English-Test was obtained from http://www.mocatest.org, the version was translated by Chinese linguists and medical specialists) was used to avoid the learning effects that can occur over short periods of time. On day 30 after surgery, patients’ cognitive status was assessed by post-discharge follow-up phone calls, and the scores were measured using the telephone version of the MoCA test. Likewise, the MoCA test Mandarin version 7.3 (the translated version of MoCA-8.3-English-Test from http://www.mocatest.org by Chinese linguists and medical specialists) was used to avoid learning effects. When the score decreased by > 1.96 points on the POD 4, the patient was considered to have delayed neurocognitive recovery [11]. Similarly, when the t-MoCA score decreased by > 1.96 points at 1 month postoperatively, the patient was considered to have delayed neurocognitive recovery.

### Data collection

The collected data included: (1) demographics and clinical baseline data, including age, gender, body mass index (BMI, kg/m^2^), comorbidities, years of education, and ASA classification; (2) main clinicopathological parameters, including blood loss, urine volume, intraoperative hypotension (MAP ≤ 65mmHg), whether midazolam and dexmedetomidine were used during anesthesia induction, type of anesthesia, anesthesia time, operation time, operation type, surgical resection, VAS score (1st day after surgery, resting, coughing and activity), analgesic rescue, chest tube duration, postoperative fever (≥ 38 °C), postoperative complications, and postoperative hospital stay; (3) laboratory tests, including the blood cell analysis which was carried out 1 day before surgery and 1 day after surgery, albumin (Alb), hemoglobin, creatinine levels, white blood cell (WBC) levels, neutrophil-to-lymphocyte ratio (NLR), and c-reactive protein (CRP) levels.

### Statistical analysis

Data were statistically analyzed using R Studio (version 4.2.1 [2022-06-23]) and reported as median (interquartile range) or odds ratio OR (95% confidence interval). Pearson’s chi-square test or Fisher’s test was used to compare dichotomous variables between groups; continuous variables that conformed to normal distribution were analyzed using two independent samples t-test, and continuous data that did not obey normal distribution were analyzed using Mann–Whitney U test. By performing a univariate logistic analysis of risk factors for DNR on postoperative day 4 and 30, we grouped participants according to the presence or absence of DNR. Factors with P value < 0.2 in the univariate analysis were included in a stepwise multivariate logistic regression analysis to identify independent risk factors for DNR in the early postoperative period and 30 days postoperatively. The univariate and multivariate analysis results were expressed as OR values ​​and 95% confidence intervals. P < 0.05 was considered as statistical significance.

## Results

### Baseline characteristics

**Fig. 1:**
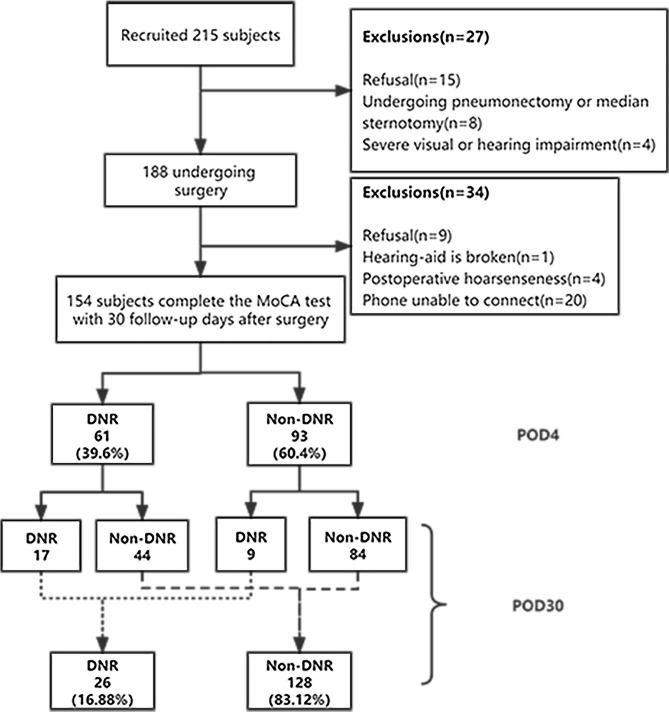
**Study flowchart.** DNR, Delayed Neurocognitive Recovery; MoCA, Montreal Cognitive Assessment; POD, postoperative days

Among 215 recruited patients, 154 of them, including 68 males (44.2%) and 86 females (55.8%), completed the 30-day postoperative cognitive follow-up (Fig. 1). The patients were divided into DNR and Non-DNR groups according to whether neurocognitive recovery delay occurred on POD4 and POD30. All patients were between 60 and 79 years old, with an average age of 67.99 ± 3.88 years and a preoperative MoCA score of 24.68 ± 2.75 points.

Baseline demographic, preoperative, intraoperative, and pathologic data of patients in the two groups, such as gender, age, BMI, years of education, smoking history, comorbidities (hypertension, hyperlipidemia, cerebrovascular events, diabetes, coronary heart disease), preoperative pulmonary infection, history of major surgery, pathological properties (malignant/benign), ASA classification, surgical type (thoracotomy or thoracoscopy), surgical resection range, operation time, anesthesia time, urine output, and blood loss were not significantly different (all P > 0.05, Table 1). However, statistically significant differences between the two groups were observed in cerebrovascular events (P = 0.003), diabetes (P = 0.033), and history of major surgery (P = 0.031).

**Table 1:** Comparison of demographics and clinical data between DNR and non-DNR groups at 30 days after surgery

Item	DNR(n = 26)	Non-DNR(n = 128)	P value
Age (years)	68(65.75–70.25)	67(65–70)	0.904
Sex (Male/Female)	11/15	57/71	0.835
Body mass index, kg/m^2^	23.25 ± 3.07	24.61 ± 3.28	0.863
Education(years)	9(8–12)	9(7–12)	0.897
Smoking	10(38.46%)	46(36.72%)	0.867
ASA status			0.704
I	0	2(1.56%)	
II	23(88.46%)	103(80.47%)	
III	3(11.54%)	23(19.53%)	
**Medical history**			
Hypertension	12(46.15%)	71(55.47%)	0.385
Diabetes mellitus	9(34.62%)	21(16.41%)	0.033*
Hyperlipidemia	2(7.69%)	11(8.59%)	1.000
History of cerebrovascular accident	8(30.77%)	10(7.81%)	0.003*
Coronary artery disease	5(19.23%)	22(17.19%)	1.000
previous major surgery history	7(26.92%)	12(9.38%)	0.031*
Preoperative pulmonary infection	5(19.23%)	33(25.78%)	0.480
Malignant/benign disease	21(80.77%)	116(90.63%)	0.263
**Surgical data**			
Surgical approach			0.87
Open surgery	3(11.53%)	17(13.28%)	
Thoracoscopic surgery	26(89.66%)	108(86.4%)	
Resection range			0.532
Non-anatomic wedge resection	0	6(4.69%)	
Segmentectomy	6(23.08%)	17(13.28%)	
Sublobar resections	7(26.92%)	37(28.91%)	
Lobectomy	10(38.46%)	53(41.41%)	
Pulmonary bilobectomy	3(11.54%)	13(10.16%)	
Anesthesia time(min)	189(152–245)	184(152–230)	0.931
Operation time(min)	118(83–184)	122(89–160)	0.944
Urine(ml)	200(100–400)	200(100–325)	0.336
Blood loss (ml)	50(20–100)	50(20–100)	0.669
Chest tube duration	3(3–4)	4(3–5)	0.189
Paravertebral block with catheter use	8(30.77%)	39(30.47%)	0.976

Abbreviations: ASA American Society of Anesthesiologists, BMI Body mass index, DNR Delayed Neurocognitive Recovery, NRS Numeric Rating Scales

Continuous variables are presented as median [IQR] or mean ± SD, and categorical variables as count (%).

*P < 0.05.

There were no significant differences between the two groups in a preoperative blood test, demographics and clinical data, as well as postoperative pain score, analgesic rescue, chest tube duration, and postoperative fever (all P > 0.05), while there were statistically significant differences in perioperative inadvertent hypothermia (< 35℃) (P = 0.041). Although there was a statistically significant difference between the two groups at the WBC level (P = 0.02), it was deemed to have no clinical significance by the study physicians (Table 2).

**Table 2:** Main clinical data and laboratory tests associated with DNR in elderly patients who underwent a major thoracic surgery

Item	DNR(n = 26)	Non-DNR(n = 128)	P value
Preoperative blood tests			
Hemoglobin < 110	1(3.85%)	8(6.25%)	1.000
Creatinine clearance (ml/min)	63.50(59.35–72.85)	65.10(56.98–78.08)	0.54
Pre-WBC levels (× 10^9^ /L)	4.35(3.84–5.11)	5.96(4.92–7.06)	0.02*
Post-WBC levels (×10^9^ /L)	12.47(9.89-17.00)	14.07(11.69–16.66)	0.243
WBC gap	7.73(5.37–11.40)	7.92(5.45–10.52)	0.887
NLR	1.98(1.41–2.38)	2.00(1.44–2.61)	0.771
Hb levels	135.00(127.50-141.50)	132(125–139)	0.259
Albumin (g/L)	40.3(38.80–43.40)	40.70(38.30–43.20)	0.871
CRP ≥ 5	2(7.69%)	13(10.16%)	0.981
**Surgical data**			
Perioperative inadvertent hypothermia(<35℃)	8(30.77%)	16(12.5%)	0.041*
Blood loss			0.659
<200	23(88.46%)	117(91.41%)	
200–500	2(7.69%)	9(7.03%)	
>500	1(3.85%)	2(1.56%)	
Hypotension(MAP < 65mmHg)	6(23.08%)	32(25.00%)	0.836
VICA(use sevoflurane ≥ 50 min)	5(19.23%)	12(9.38%)	0.144
Midazolam as a pre-anaesthetic sedative	16(61.54%)	94(73.44%)	0.221
Dexmedetomidine	21(80.77%)	113(88.28%)	0.472
Rescue analgesia			
Opium analgesic	10(38.46%)	56(43.75%)	0.619
Ketorolac Tromethamine	17(65.38%)	86(67.19%)	0.859
NRS			
1st day after operation(resting)			0.792
<4	22(84.62%)	111(86.72%)	
4–6	4(15.38%)	16(12.50%)	
>6	0	1(0.78%)	
1st day after operation(activity)			
<4	14(53.85%)	72(56.25%)	0.730
4–6	12(46.15%)	51(39.84%)	
>6	0	5(3.91%)	
1st day after operation(coughing)			0.364
<4	9(34.62%)	57(44.53%)	
4–6	16(61.54%)	69(53.91%)	
>6	1(3.85%)	2(1.56%)	
Chest tube duration ≥ 4 days	10(38.46%)	68(53.13%)	0.173
Postoperative hospital stay(days)	7(6–9)	7(6–8)	0.723
Postoperative body temperature > 38 ℃	4(15.38%)	38(29.69%)	0.135

Abbreviations: DNR Delayed Neurocognitive Recovery, NRS Numeric Rating Scales, NLR Neutrophil-to-lymphocyte Ratio, VICA Intravenous inhalation combined anesthesia

Continuous variables are presented as median [IQR] or mean ± SD, and categorical variables as count (%).

*P < 0.05.

### Risk factors associated with DNR 30 days after surgery

**Fig. 2:**
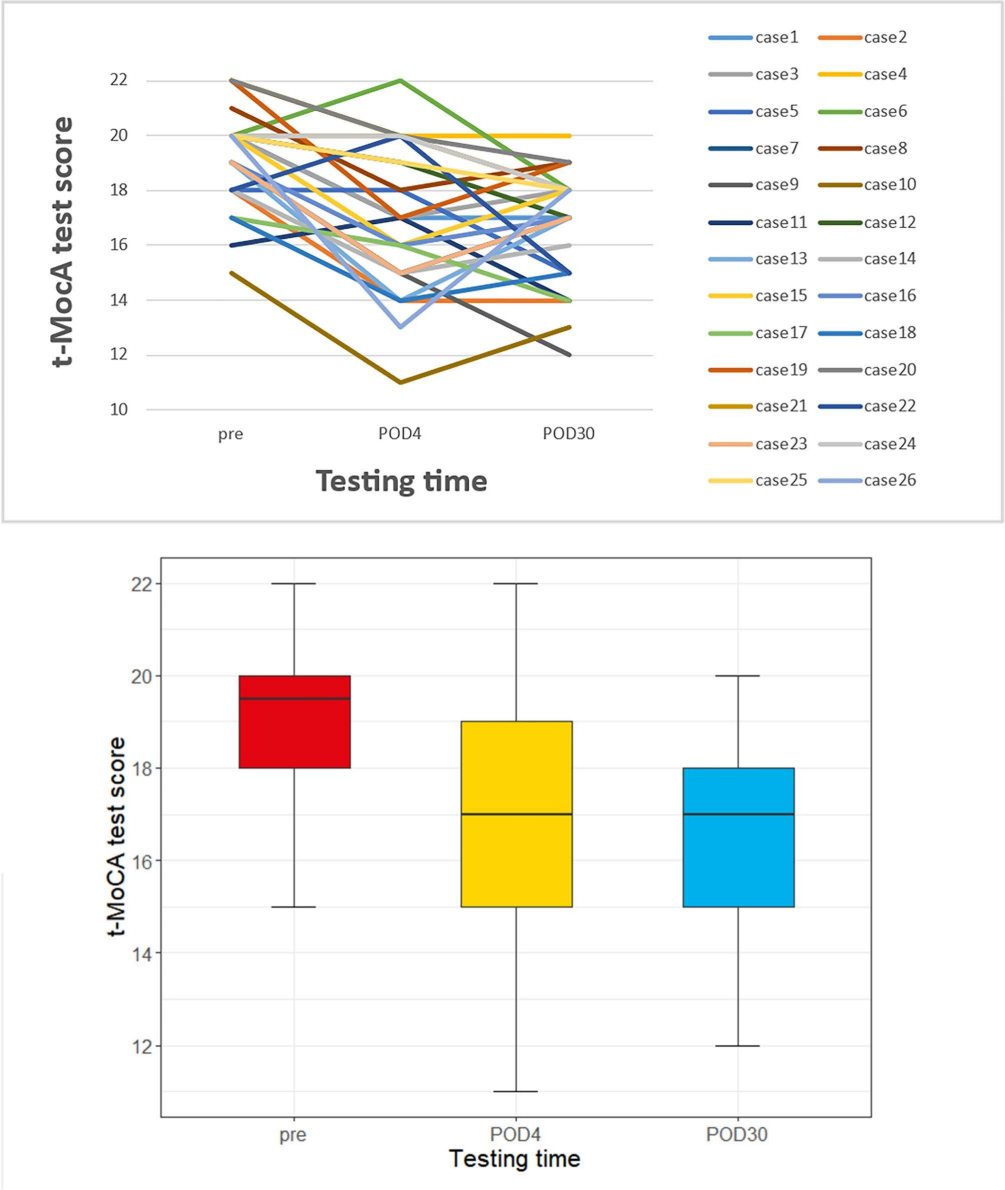
Change in T-MoCA Scores in patients with DNR at POD30.

On POD30, 26 of the 154 patients developed DNR (16.88%). Among these 26 patients with DNR, 17 (17/61, 27.87%) had delayed postoperative cognitive recovery on the POD4 and 9 patients had newly emerging DNR, while 128 (128/154, 83.12%) patients had cognitive recovery corresponding to the baseline levels. The t-MoCA scores of the 26 patients with DNR changed at three-time points (Fig. 2). The t-MoCA scores of the 26 patients were 19.5 (18–20) before surgery, 17 (15-19.25) on POD4, and 17 (15–18) on POD30. Among 26 patients with DNR at POD30, only 5 patients had no cognitive decline on POD4, and the remaining 21 patients had varying degrees of score decrease on POD4 (Fig. 2).

Univariate Logistic regression analysis showed that a history of diabetes mellitus (OR = 2.697 [1.060–6.861], P = 0.037), history of stroke (OR = 5.244 [1.828–15.042], P = 0.002), intraoperative hypothermia (≤ 35 ℃, lasting ≥ 30 min) (OR = 3.111 [1.163–8.322], P = 0.024), VICA (OR = 2.302 [0.735–7.212], P = 0.153) and history of major surgery (OR = 3.561 [1.245–10.185], P = 0.018) were potential risk factors for DNR on POD30 in elderly patients who underwent thoracic surgery. In addition, statistics showed that a history of diabetes (OR = 6.508 [2.049–20.664], P = 0.001), intraoperative hypothermia (≤ 35 ℃, lasting ≥ 30 min)(OR = 5.688 [1.693–19.109], P = 0.005), history of cerebrovascular events (OR = 10.211 [2.842–36.688], P < 0.001), and VICA (using sevoflurane combined with propofol anesthesia) (OR = 5.306 [1.272–22.138], P = 0.022) were independent risk factors for DNR (Table 3).

**Table 3:** Univariate logistic regression analysis of risk factors for delayed cognitive recovery at 30 days

	Univariate analysis		Multivariate analysis	
**Risk factors**	OR(95%CI)	P value	OR(95%CI)	P value
Age(age ≥ 70, age<70)	0.841(0.327–2.163)	0.719		
Smoking	1.077(0.452–2.566)	0.867		
**Medical history**				
Diabetes mellitus	2.697(1.060–6.861)	0.037**	6.508(2.049–20.664)	0.001**
History of cerebrovascular disease	5.244(1.828–15.042)	0.002**	10.211(2.842–36.688)	<0.001**
Previous major surgery history	3.561(1.245–10.185)	0.018**		0.065*
**Surgical data**				
VICA(Sevoflurane combined with propofol was used for anesthesia)	2.302(0.735–7.212)	0.153*	5.306(1.272–22.138)	0.022**
Hypotension(MAP ≤ 65mmHg, ≥5 min)	0.900(0.332–2.437)	0.836		
Perioperative inadvertent hypothermia (< 35℃)	3.111(1.163–8.322)	0.024**	5.688(1.693–19.109)	0.005**
Pre-NLR ≥ 2.5	0.797(0.296–2.149)	0.654		
NRS (resting) ≥ 4 at 1st day after surgery	1.187 (0.364–3.869)	0.776		

Abbreviations: DNR Delayed Neurocognitive Recovery, NRS Numeric Rating Scales, NLR Neutrophil-to-lymphocyte Ratio, VICA Intravenous inhalation combined anesthesia

*P < 0.2, ** P < 0.05.

### Risk factors associated with DNR on postoperative day 4

On the 4th postoperative day, 61 (39.6%) had DNR among the 154 patients. The results of univariate logistic regression analysis showed that a history of diabetes mellitus (OR = 2.006 [0.897–4.489], P = 0.09), patient age ≥ 70 years old (OR = 1.842 [0.915–3.710], P = 0.087), preoperative peripheral blood neutrophil-lymphocyte ratio (NLR) ≥ 2.5 (OR = 0.462 [0.211–1.012], P = 0.054), and history of major surgery (OR = 2.337 [0.881-6.200], P = 0.088) could be risk factors for early postoperative DNR. Factors with P < 0.2 were included in multivariate regression, and the results showed that age ≥ 70 years (OR = 2.311 [1.096–4.876], P = 0.028) and preoperative NLR ≥ 2.5 (OR = 0.428 [0.188–0.975], P = 0.043) were independent risk factors for DNR on POD4 (Table 4).

**Table 4:** Univariate logistic regression analysis of risk factors for delayed cognitive recovery on the 4th day

	Univariate analysis		Multivariate analysis	
**Risk factors**	OR(95%CI)	P value	OR(95%CI)	P value
Age (age ≥ 70, age<70)	1.842(0.915–3.710)	0.087*	2.311(1.096–4.876)	0.028**
Smoking	0.831(0.424–1.630)	0.59		
**Medical history**				
Diabetes mellitus	2.006(0.897–4.489)	0.09*	1.972(0.845–4.599)	0.116
History of cerebrovascular disease	1.615(0.602–4.332)	0.341		
Previous major surgery history	2.337(0.881-6.200)	0.088*	2.126(0.764–5.918)	0.149
**Surgical data**				
VICA(use sevoflurane ≥ 50 min)	1.409(0.512–3.878)	0.507		
Hypotension(MAP ≤ 65mmHg)	1.147(0.545–2.416)	0.717		
Perioperative inadvertent hypothermia(<35℃)	1.354(0.563–3.255)	0.498		
Pre-NLR ≥ 2.5	0.462(0.211–1.012)	0.054*	0.428(0.188–0.975)	0.043**
NRS (resting) ≥ 4 at 1st day after surgery	0.731(0.277–1.932)	0.528		

Abbreviations: DNR Delayed Neurocognitive Recovery, NRS Numeric Rating Scales, NLR Neutrophil-to-lymphocyte Ratio, VICA Intravenous inhalation combined anesthesia

*P < 0.2, ** P < 0.05.

## Discussion

The present study detected a significant relationship between DNR and previous cerebrovascular events, history of diabetes, VICA, and intraoperative hypothermia. Therefore, the prevention of DNR in surgical patients has positive clinical guiding significance. Our results showed that 40% of elderly patients undergoing thoracic surgery were affected by DNR on POD4, and DNR was still present in 16.88% of patients on POD30.

According to previous studies, potential risk factors [2, 7] for DNR after major surgery are advanced age, preoperative comorbidities (diabetes, hypertension, previous cerebrovascular events, etc.), smoking, unplanned surgery, postoperative analgesia [12] and intraoperative hypotension [13], etc. Herein, we evaluated the risk factors in these patients at high risk for DNR and found that a history of diabetes, perioperative inadvertent hypothermia, previous cerebrovascular events, and VICA were independent risk factors for the occurrence of DNR on POD30. We also found that age ≥ 70 was an independent risk factor for DNR on POD4. In addition, a previous study revealed that NLR ≥ 2.5 was a risk factor for DNR [14], which was further confirmed study by Li et al. [15]. In the present study, we also found that preoperative NLR ≥ 2.5 was an independent risk factor for DNR on POD4.

In this study, ≥ 70 years of age at POD4 resulted as an independent risk factor for the occurrence of DNR in patients. Similarly, it was noted that aging was associated with neuroinflammation and that peripheral immune responses to surgical trauma lead to excessive neuroinflammation in the aged brain. The older the surgical patient, the greater the likelihood of postoperative cognitive dysfunction [16, 17], which is consistent with the results of the present study.

We also found that preoperative NLR ≥ 2.5 was an independent risk factor for delayed neurocognitive recovery on POD 4. This is consistent with Yong’s finding, suggesting that NLR ≥ 2.5 predicted DNR in gastric cancer patients [14]. Hadi et al. also revealed that NLR was associated with a 3-fold increase in the risk of DNR 1 day after carotid endarterectomy [18]. NLR is a circulating biomarker of systemic inflammation frequently used as a predictor of adverse outcomes in tumor-related studies [19, 20], and the mechanism through which elevated NLR levels are associated with DNR remains unclear. Thus far, inflammation has received increasing attention as a potential mechanism of perioperative cognitive impairment [21, 22]. Thoracic surgery often requires one-lung ventilation with severe physiological disturbances and over-activation of inflammatory processes. There is substantial evidence that elevated levels of inflammation negatively affect cognitive abilities, including attention, memory, and executive function [23, 24]. However, neither age ≥ 70 years nor preoperative NLR ≥ 2.5 resulted as risk factors for DNR at POD30. The relatively conservative scope of surgery in the senior surgical population is thought to be related to the patients’ neurological inflammatory system response having recovered by POD30. The hit of systemic inflammatory response caused by surgery and the neuroinflammation in the fragile brains of older people has become relieved one month after surgery. Thus, NLR ≥ 2.5 or age ≥ 70 is an independent risk factor of DNR at POD4 rather than POD30.

Diabetes mellitus and its complications have become a major public health concern, as well as an economic burden in many countries [25, 26]. In our study, 19.5% of elderly patients undergoing thoracic surgery had diabetes before surgery. Diabetes is considered a chronic inflammatory state, and several studies have found a significant relationship between diabetes and DNR [27, 28]. In their study, Stratton et al. [29] pointed out that complications in patients with type 2 diabetes, such as macrovascular and microvascular disease, and poor perioperative blood glucose control, may lead to cerebrovascular microcirculatory disturbances and adversely affect postoperative cognitive function. In addition, a previous study showed that high preoperative levels of glycated hemoglobin in diabetic patients were correlated with poor postoperative cognitive test scores [30]. In this study, we confirmed that diabetes was an important risk factor for DNR.

Our results revealed that previous cerebrovascular events were an independent risk factor for DNR. Some studies have shown that previous cerebrovascular events are a risk factor for cognitive adverse events [31], which is consistent with the present study’s findings and may be related to the fact that stroke is often associated with cerebral oxygen desaturation. In addition, we speculate that intraoperative impairment of cerebrovascular autoregulation is associated with postoperative neurocognitive function early after oncologic surgery [32, 33].

VICA (sevoflurane combined with propofol anesthesia) was an independent risk factor for DNR in our research. Although some scholars believe that sevoflurane is not the reason for PND. Walters et al. [34] found that sevoflurane exposure had little effect on the cognitive function of adult rhesus monkeys and did not alter microglia activation. A clinical study by Schoen et al. [35] revealed that patients receiving sevoflurane anesthesia performed better than those receiving propofol in the abbreviated mental test, Stroop test, trail-making test, word Lists, and mood-assessment tests. However, Zhang and colleagues [36] found an increased risk of DNR with sevoflurane compared to propofol anesthesia. They speculated that sevoflurane may have neurotoxic effects through the accumulation of β-amyloid and increase the expression of inflammatory cytokines. Also, higher postoperative pain scores in patients with inhaled anesthesia had a negative effect on cognition. A study found that sevoflurane anaesthesia compared to anesthesia with propofol, might increase the incidence of delayed neurocognitive recovery in older adults after major cancer surgery [4]. Interestingly, although univariate analysis revealed no significant difference in the use of sevoflurane anesthesia in the present study, its inclusion in the multivariate analysis showed that the application of sevoflurane anesthesia was an independent risk factor for DNR. Accordingly, we speculated that the true effectiveness of sevoflurane negative effects on cognition might be masked in the univariate analysis by the effects of propofol on neurocognition, diabetes, previous cerebrovascular events, and other risk factors. This may explain why the use of VICA was significant in the multivariate analysis but not in the univariate analysis.

We found that intraoperative hypothermia (≤ 35 °C for ≥ 30 min) was a risk factor for perioperative cognitive impairment, which is consistent with previous findings [37]. However, a recent systematic review [13] reported no significant difference in the rates of DNR when comparing normothermic with hypothermic cardiopulmonary bypass (CPB) (0.55 [0.47–0.62] vs. 0.58 [0.47–0.69], respectively). Only 7 of 35 trials (20%) assessing DNR were RCTs for target temperature management during CPB, and the lack of appropriately controlled trials may be one of the main factors leading to the different conclusions. Another study found that warming blood transfusion and infusion could significantly reduce the occurrence of perioperative hypothermia while having no significant effect on postoperative recovery of cognitive. Also, the link between DNR and intraoperative hypothermia was not explored in that study [38].

The present study has some limitations. This is a single-center observational study with a limited number of cases. We only investigated the clinical characteristics of patients and did not conduct imaging examinations and molecular mechanism studies. Future large sample multicenter prospective studies, combining imaging examinations and new molecules to predict the occurrence of DNR in the elderly, expanding the research scale, and applying more standardized methods are warranted. Besides, we did not observe a relationship between postoperative pain and DNR, which we hypothesized was due to the confounding of patients receiving paravertebral analgesia and opioid intravenous analgesia. For high-risk patients, the following targeted interventions can be taken: preoperative assessment, improvement of general condition, advance prediction of surgical difficulty and risk, intraoperative maintenance of circulatory stability, intraoperative cerebral oxygen saturation monitoring, intraoperative warming, active perioperative period blood sugar management measures, multimodal postoperative analgesia, etc.

## Conclusions

Elderly patients with thoracic surgery were found to have a higher incidence of DNR, while the preoperative history of diabetes, history of cerebrovascular events, VICA (using sevoflurane combined with propofol anesthesia), and intraoperative hypothermia (≤ 35 °C, lasting ≥ 30 min) resulted as independent risk factors for DNR on POD30. Overall, it is likely that absent major physiological perturbations, some perioperative management choices might have a minor impact on postoperative neurocognitive outcomes. Larger prospective studies are needed to further investigate DNR’s risk factors and prognosis in the elderly population.

## Data Availability

All data generated or analysed during this study are included in this published article.
